# A dataset of barometric readings for enhancing security and privacy of IoT

**DOI:** 10.1016/j.dib.2023.109782

**Published:** 2023-11-07

**Authors:** Omar Adel Ibrahim, Gabriele Oligeri, Roberto Di Pietro

**Affiliations:** a RC3, CEMSE Division, King Abdullah University of Science and Technology (KAUST), Thuwal, Saudi Arabia; b Division of Information and Computing Technology, College of Science and Engineering, Hamad Bin Khalifa University, Doha, Qatar

**Keywords:** Contextual security, Context-based pairing, Pressure-based authentication, Zero-interaction, IoT, Proximity-based authentication

## Abstract

The security and privacy of wireless channels is typically enforced by leveraging cryptographic tools. However, there are scenarios where these methods are unfit, such as in resource-constrained environments, i.e., Internet of Things (IoT), or when an extra layer of security is needed. A promising solution involves correlating air pressure (barometric) readings to securely pair IoT devices while requiring zero-interaction. This paper presents an experimental dataset of real-world barometric measurements collected in open areas under different weather conditions. Specifically, our dataset includes readings recorded using the reference hardware platform BMP280. The experiments involve a reference scenario constituted by three Adafruit BMP280 barometric sensors connected to a Raspberry Pi 3 Model B board to collect barometric measurements. The three sensors represent two communicating parties (Alice and Bob) and an adversary (Eve), respectively. The dataset is constituted by three experiments characterized by different relative distances among Alice, Bob, and Eve. We considered 5cm and 2m between Alice and Bob while placing Eve at 2m and 8 meters, respectively. The second configuration, i.e., (Alice-Bob at 2m and Eve at 8m) has been replicated in a different scenario characterized by less air pressure fluctuations. The sampling frequency has been set to 70Hz while the measurements last for 50, 24 and 41 hours, respectively.

Researchers can use this dataset in several ways, including: (i) Study the air pressure variation and correlation between devices separated by different distances, (ii) Develop a co-location verification extension for the Diffie-Hellman (DH) key agreement method that utilizes air pressure data streams, (iii) Study possible attacks against proximity-based authentication techniques that depend on pressure correlated variations.

Specifications Table

**Table:** 

Subject	Computer Science: Computer Networks and Communications
Specific subject area	IoT authentication and co-location verification extension to Diffie-Hellman (DH) key agreement method using out-of-band (OOB) auxiliary channels
Data format	Raw data.
Type of data	Table
Data collection	We acquired the data by conducting real measurements in an outdoor environment, using three Adafruit BMP280 pressure sensors connected to a Raspberry Pi 3 Model B board . We used a Python script run on the Raspberry Pi to collect the air pressure data from the three connected BMP280 sensors while saving the readings into .CSV files. The dataset contains barometric readings in Pascal (Pa) unit for 3 parties: Alice, Bob and Eve, each in a separate column while each row represents a specific time instance. We considered a sampling frequency of 70 Hz, i.e., 70 rows represent one second of measurements. Overall, we have 3 traces: Total of more than 110 hours. (i) First Scenario: 50 hours, windy periods, 5cm separating Alice and Bob, with Eve at 2m distance. (ii) Second Scenario: 24 hours measurement where Alice and Bob are separated by 2m, with Eve at 8m. (iii) Third Scenario: 41-hours long trace in a quiet environment with low air pressure variations, Alice and Bob are separated by 2m, with Eve at 8m.
Data source location	Institution: Hamad Bin Khalifa University City/Town/Region: Doha Country: Qatar Latitude and longitude (and GPS coordinates, if possible) for collected samples/data: [25.31557, 51.43446]
Data accessibility	Repository name: Mendeley Data Data identification number: 10.17632/vfk4b45jnk.3 Direct URL to data: https://data.mendeley.com/datasets/vfk4b45jnk/3
Related research article	The related research article is published in 2022 IEEE Conference on Communications and Network Security (CNS) [1]: Omar Adel Ibrahim, Gabriele Oligeri, and Roberto Di Pietro. “Eolo: IoT Proximity-based Authentication via Pressure Correlated Variations.” In 2022 IEEE Conference on Communications and Network Security (CNS), pp. 109-117. IEEE, 2022. https://doi.org/10.1109/CNS56114.2022.9947258

## 1 Value of the Data

• The recorded air pressure samples enable the research community to study the possibility of co-location verification of devices equipped with pressure sensors [1].
• To the best of our knowledge, we are the first to provide such dataset [2] by collecting the barometric readings from two legitimate communicating parties (Alice and Bob) in addition to a malicious party (Eve). The 3 parties are separated by different distances and are all recorded simultaneously.
• Researchers may use the dataset to develop new authentication techniques that augment the existing multi-factor authentication schemes.
• Researchers can evaluate co-location verification capabilities in a wide range of authentication scenarios, using several different hardware devices equipped with pressure sensors, across different distances and weather conditions.

## 2 Data Description

This dataset consists of 3 files:

(i) First file: 5cmAB_2mE_50h.csv (552 MB)
  50 hours of air pressure data recording, including some windy periods, 5cm separating Alice and Bob, with Eve at 2m distance from Alice and Bob. The number of table entries is 12,600,000.
(ii) Second file: 2mAB_8mE_24h.csv (298 MB)
  24 hours of air pressure data recording, including some windy periods, 2m separating Alice and Bob, with Eve at 8m distance from Alice and Bob. The number of table entries is 6,048,000.
(iii) Third file: 2mAB_8mE_41h_Quiet.csv (509 MB)
  41 hours of air pressure data recording, quiet environment with low air pressure variations, 2m separating Alice and Bob, with Eve at 8m distance from Alice and Bob. The number of table entries is 10,332,000.

**Fig. 1:**
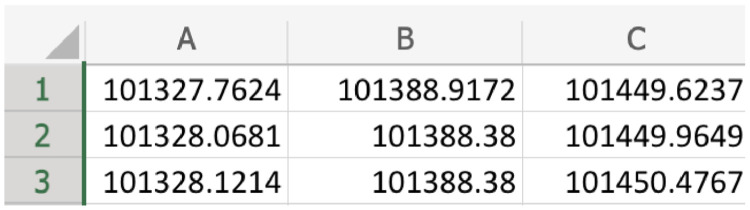
Snapshot of file structure in the dataset in Excel environment.

In each file, there are three columns, as shown in Fig. 1:

First column: Alice air pressure reading.
Second column: Bob air pressure reading.
Third column: Eve air pressure reading.

All air pressure readings are recorded in Pascal (Pa) unit, and the Sampling frequency = 70 Hz, so every 70 rows represent one second measurement of air pressure data recorded simultaneously at a specific time instance from the 3 parties (Alice, Bob and Eve).

## 3 Experimental Design, Materials and Methods

An example analysis and a use case of the dataset presented in this paper is detailed in [1]. In the following, we focus on the hardware and software setups.

### 3.1 Hardware setup

To gather data of air pressure variations, we used three Adafruit BMP280 pressure sensors connected to a Raspberry Pi 3 Model B board. The sensors were used to represent two communicating parties, Alice and Bob, and an adversary, Eve. To ensure better synchronization, the sensors were connected to the same Raspberry Pi board. The experiments were carried out in an outdoor open area, with Alice and Bob initially placed 5cm apart, then 2m apart, and Eve positioned 2m away from Alice and Bob, then 8m away. This was done to cover various scenarios and distances. All sensors are within Line-of-sight (LOS) of each others.

Raspberry Pi 3 Model B is a single board computer that is well known for its compact size and affordability. It is equipped with a quad core CPU running at 1.2 GHz, 1 GB of RAM, built in WiFi and Bluetooth connectivity. In addition, it offers a wide range of interfaces including HDMI, USB ports, GPIO pins and a microSD card slot. The board is used in many applications especially Internet of Things (IoT) projects.

The BMP280 sensor is a compact and precise digital sensor that measures atmospheric pressure and temperature. The sensor communicates with microcontrollers using digital interfaces such as I2C or SPI. BMP280 sensors are known for their high accuracy, making them suitable for applications such as weather monitoring, altitude tracking, and indoor environmental sensing. They are widely used in IoT projects and weather-related applications due to their reliability and real-time data output capabilities.

To connect three BMP280 sensors to a Raspberry Pi 3 Model B, we use the I2C (Inter-Integrated Circuit) interface since the BMP280 sensors typically communicate over I2C. Note that Each BMP280 sensor must have a unique I2C address to avoid conflicts since they share the same bus.

On a Raspberry Pi 3 Model B, the I2C interface pins and their connections to the BMP280 sensors are as follows:

­ SDA (Serial Data): GPIO2 (Pin 3). It is connected to the SDI pin on BMP280 sensor.
­ SCL (Serial Clock): GPIO3 (Pin 5). It is connected to the SCK pin on BMP280 sensor.
­ 3.3V Power: Pin 1 (3.3V). It is connected to the VIN pin on BMP280 sensor.
­ Ground (GND): Pin 6 (GND). It is connected to the GND pin on BMP280 sensor.

We can basically use any GPIO pins of the Raspberry Pi to connect the SDA and SCL lines of the BMP280 sensors, provided they are available. Each BMP280 sensor should have a unique combination of SDA and SCL pins, and each sensor should have a distinct I2C address.

**Fig. 2:**
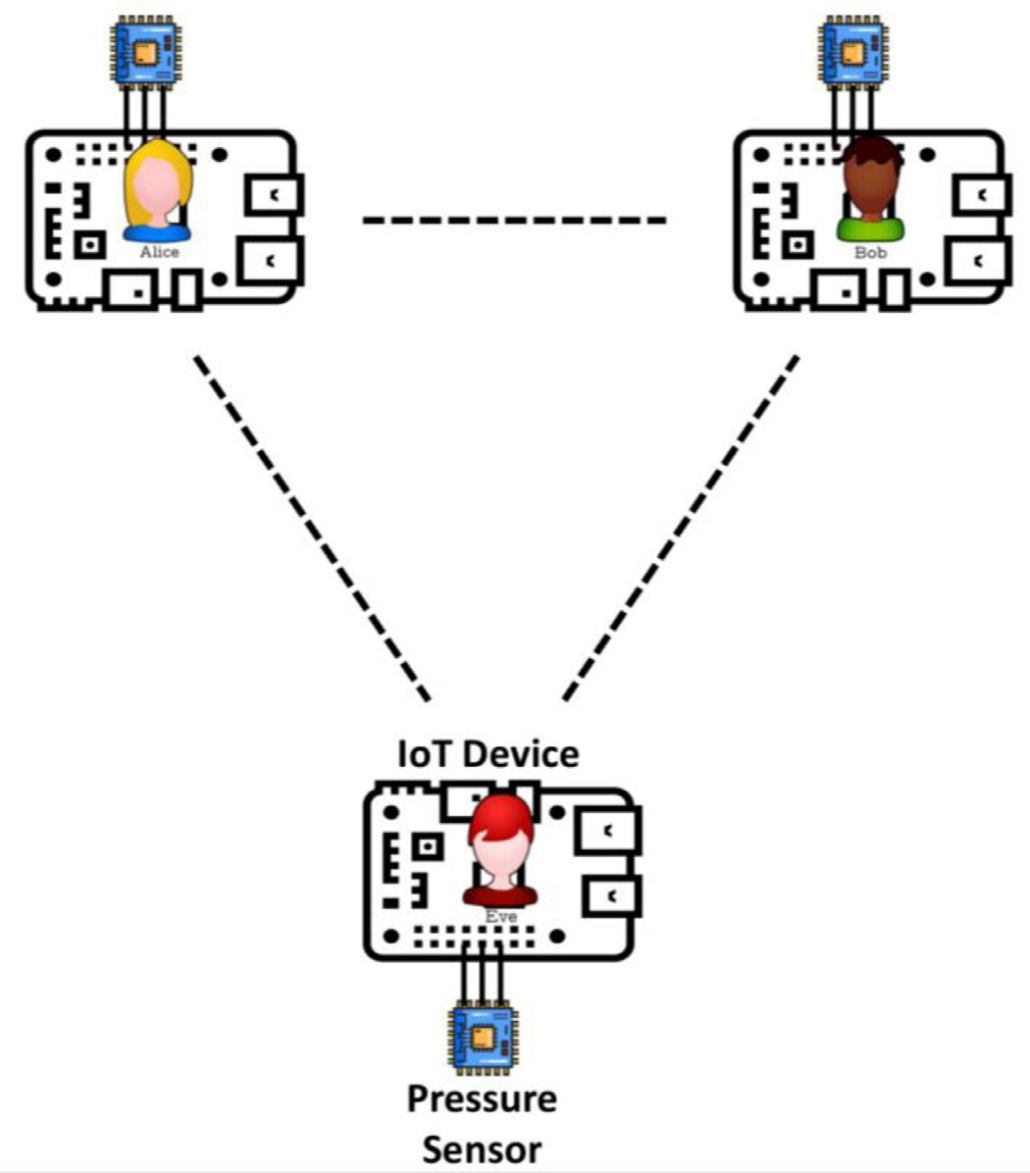
Hardware Deployment Setup for dataset collection.

The hardware deployment setup is illustrated in Fig. 2.

### 3.2 Software setup

We used a simple Python code run on the Raspberry Pi to read the air pressure sample from the three connected BMP280 pressure sensors and save it to .CSV files. Sample code and BMP280 sensor connection details can be found in [3]. We wrote a Python script that communicates with the BMP280 sensors using their respective I2C addresses as follows:

­ Import the required libraries (mainly Adafruit BMP280 library).
­ Initialize and read data from each BMP280 sensor separately by specifying its address.
­ Execute the Python script on the Raspberry Pi to read data from the BMP280 sensors.

By following these steps, we are able to connect and interface with three BMP280 sensors on your Raspberry Pi 3 Model B over the I2C bus. This setup allows the collection of atmospheric pressure data from multiple sensors simultaneously. While all the three sensors are linked to the same Raspberry Pi board and undergo simultaneous sampling for better synchronization, it is also possible to connect each sensor to a separate microcontroller and record the timestamp of each sample to match corresponding samples from different devices.

## Limitations

The dataset needs to be expanded to include more different conditions, such as: rainy environment, Non-line-of-sight (NLOS) between sensors, and indoor scenarios. It would also be beneficial to replicate this dataset using different devices equipped with barometers (e.g., Mobile phones). Such extension to the current dataset would allow for a more robust analysis of the variation in barometric readings between different recording devices in different conditions, and opens the door for researchers to investigate different novel techniques for co-location verification and augment muti-factor authentication frameworks.

## Ethics Statements

The current work does not involve human subjects, animal experiments, or any data collected from social media platforms.

## CRediT authorship contribution statement

**Omar Adel Ibrahim:** Conceptualization, Methodology, Software, Validation, Investigation, Resources, Data curation, Writing – original draft. **Gabriele Oligeri:** Conceptualization, Methodology, Software, Validation, Investigation, Resources, Data curation, Writing – original draft. **Roberto Di Pietro:** Conceptualization, Methodology, Software, Validation, Investigation, Resources, Data curation, Writing – original draft.

## Data Availability

A Dataset of Barometric Readings for Enhancing Security and Privacy of IoT (Original data) (Mendeley Data)
